# Mucoadhesive Gel Based on *Cinnamomum cassia* Essential Oil and *Punica granatum* Extract for Oral Candidiasis: A Pilot Study of Plant Synergism

**DOI:** 10.3390/pharmaceutics18080914

**Published:** 2026-07-24

**Authors:** Naira Neri Santana, Larissa Aparecida Engel, Maísa Steffani Adamczuk, Talita Anielle de Assunção, Mariana Dalmagro, Daniella Londero Silva Batisti, Paula Derksen Macruz, Eduardo Jorge Pilau, Lidiane Nunes Barbosa, Odair Alberton, Jaqueline Hoscheid

**Affiliations:** 1 Professional Master’s Program in Medicinal Plants and Herbal Medicines in Primary Care, University of Paraná, Umuarama 87502-210, Paraná, Brazil; naira51084@edu.unipar.br (N.N.S.); lidianebabosa@prof.unipar.br (L.N.B.); odair@prof.unipar.br (O.A.); 2 Postgraduate Program in Biotechnology Applied to Agriculture, University of Paraná, Umuarama 87502-210, Paraná, Brazil; larissa.engel@edu.unipar.br (L.A.E.); mariana.dal@edu.unipar.br (M.D.); 3 Postgraduate Program in Pharmaceutical Sciences, State University of Maringá, Maringá 87080-000, Paraná, Brazil; maisa.adamczuk@edu.unipar.br; 4 Pharmacy Academy, University of Paraná, Toledo 85903-170, Paraná, Brazil; talita.assuncao@edu.unipar.br; 5 Postgraduate Program in Animal Science with an Emphasis on Bioactive Products, University of Paraná, Umuarama 87502-210, Paraná, Brazil; daniella20477@edu.unipar.br; 6 Department of Chemical Engineering, State University of Maringá, Maringá 87080-000, Paraná, Brazil; pauladmacruz@gmail.com; 7 Laboratory of Biomolecules and Mass Spectrometry, Department of Chemistry, State University of Maringá, Maringá 87080-000, Paraná, Brazil; ejpilau@uem.br

**Keywords:** *Candida albicans*, checkerboard, cinnamon, denture stomatitis, pomegranate

## Abstract

**Background/Objectives:** Oral candidiasis is one of the most prevalent infections among denture wearers and is frequently associated with colonization by *Candida albicans*. In this context, natural products have been investigated as alternative therapeutic strategies, particularly *Punica granatum* peel extract and *Cinnamomum cassia* essential oil, which are recognized for their antimicrobial activity. This study aimed to develop and evaluate a mucoadhesive gel loaded with *P. granatum* and *C. cassia* for the treatment of oral candidiasis. **Methods:** The synergistic potential between the plant materials was assessed using the checkerboard method against *C. albicans*, and the inhibitory concentration was used as a parameter for the development of the mucoadhesive formulation. The gel was characterized and subsequently evaluated in denture-wearing patients with clinical manifestations of oral candidiasis for 14 days, with the results compared to treatment with nystatin. **Results:** The combination of plant materials exhibited a synergistic effect, with an inhibitory concentration of 0.1953 mg mL^−1^ for both components. Clinical evaluation demonstrated a satisfactory therapeutic response in patients treated with the gel, with resolution of symptoms at the end of the treatment period. Statistically significant advantages were also observed regarding ease of application, absence of irritation, and reduced interference with speech and swallowing. In contrast, the group treated with nystatin showed only partial clinical improvement, with additional therapy required in some cases. **Conclusions:** These findings indicate that the mucoadhesive gel represents a promising topical strategy for the management of oral candidiasis in denture wearers. However, studies with longer follow-up periods are required to consolidate the clinical evidence.

## 1. Introduction

Oral candidiasis is an opportunistic infection of the oral mucosa most caused by *Candida albicans*. This condition generally arises from an imbalance between host defense mechanisms and the resident microbiota and is frequently observed in denture wearers, as well as in immunocompromised individuals and patients with chronic conditions such as diabetes mellitus [1]. Several predisposing factors contribute to the development of this infection, including prolonged use of poorly fitted dentures, inadequate oral hygiene, xerostomia, prolonged administration of broad-spectrum antibiotics, and immunosuppression [2].

Conventional management of oral candidiasis includes topical antifungal agents, such as nystatin and miconazole, and systemic antifungals, including fluconazole, which are generally indicated for more extensive or recurrent cases [2,3,4]. However, the therapeutic effectiveness of these agents largely depends on patient adherence and on the elimination of local predisposing factors. In addition, increasing evidence indicates that treatment failures associated with commonly used antifungal drugs are mainly related to incomplete eradication of the infection and the persistence of recurrent episodes. These limitations are often associated with the formation of mature fungal biofilms, the presence of non-*albicans Candida* species with lower susceptibility to azole antifungals, and the difficulty of integrating host-related factors with prolonged therapeutic regimens [5]. Therefore, the development of alternative therapeutic strategies that combine prolonged drug delivery systems, antibiofilm activity, and integrated clinical management is of considerable interest.

In this context, the search for substances with antifungal potential has intensified scientific interest in natural products. The use of plant-derived resources for the management of various diseases has been widely investigated to explore the therapeutic potential of medicinal flora. Although medicinal plants have long been used in traditional medicine systems, their application in dentistry remains relatively underexplored, highlighting the need for further research considering the vast diversity of bioactive plant species [6].

*Cinnamomum cassia* Presl, commonly known as cinnamon, is a small tree from which essential oil (EO) can be obtained from both bark and leaves. In the food industry, its EO is often used as a natural additive due to its antimicrobial properties, which can inhibit the growth of pathogenic and spoilage microorganisms, as well as its antioxidant and antifungal activities. Its chemical composition is characterized mainly by cinnamaldehyde, cinnamyl acetate, eugenol, tannins, and sugars [7].

Another medicinal plant of significant scientific interest is pomegranate (*Punica granatum* L.), which has been traditionally consumed in various forms, including teas, juices, snacks, salads, sauces, and fresh fruit preparations. *P. granatum* is a woody shrub with small leaves and reddish-orange flowers, producing round fruits containing numerous seeds and originating from Western Asia. Its phytochemical composition includes tannins, alkaloids, sugars, terpenoids, resins, organic acids, flavonoids, and polyphenols [8].

Pomegranate has demonstrated considerable potential as an adjunct in dental therapies, particularly due to its anti-inflammatory properties in the treatment of oral mucosal disorders. Furthermore, it exhibits antioxidant, antimicrobial, anti-inflammatory, and wound-healing activities, which make it a promising natural resource for oral healthcare [9]. Its antifungal activity against *C. albicans* has been previously reported [10], while its antioxidant properties may also contribute to the management of periodontal conditions and oral inflammatory disorders [9].

Despite the well-documented antifungal properties of *P. granatum* extracts and *C. cassia* EO when evaluated individually, studies investigating their combined use remain unavailable in the current scientific literature. To the best of our knowledge, no published studies have evaluated the antifungal activity of this botanical combination, nor have they reported the development of pharmaceutical formulations incorporating both bioactive ingredients into a single delivery system. Considering that combinations of natural products may enhance therapeutic efficacy through complementary mechanisms of action, the investigation of this phytotherapeutic blend represents a novel and promising strategy for the development of adjunctive treatments for oral candidiasis.

Therefore, the present pilot study aimed to evaluate the therapeutic potential of medicinal plants in the management of oral candidiasis, focusing on the synergistic combination of cinnamon EO and pomegranate extract incorporated into a mucoadhesive system. By exploring plant-based antifungal strategies capable of targeting *Candida* biofilm formation, this approach may contribute to the development of more effective, safe, and clinically applicable therapies for a highly prevalent oral condition.

## 2. Materials and Methods

### 2.1. Plant Materials

#### 2.1.1. Acquisition of *Cinnamomum cassia* Essential Oil

The EO of cinnamon was commercially obtained from WNF Indústria e Comércio Ltda. (São Paulo, Brazil), with raw material sourced from China. According to the supplier, the EO was extracted from the bark, leaves, and stems of the plant.

#### 2.1.2. Preparation of *Punica granatum* Extract

Pomegranate fruits were collected from a rural property located in the municipality of Quatro Pontes, Paraná, Brazil (geographic coordinates: −24.594095; −53.962983). A voucher specimen was prepared for botanical identification and deposited in the Herbarium of Universidade Paranaense under code 02.

The fruits were manually depulped, and the peels were separated and dried in a forced-air circulation oven (FANEM^®^, model Iron 520, São Paulo, Brazil) at 45 °C until constant weight was achieved. Subsequently, the dried material was ground using a knife mill (Tecnal^®^, model TE-631/2, Piracicaba, Brazil) and stored under appropriate conditions until extraction.

The extraction procedure was performed according to the method described by Lima et al. [11], with minor modifications. Briefly, the extract was obtained by decoction in boiling water for 30 min using a sample-to-solvent ratio of 1:10 (*w*/*v*). After the extraction period, the decoction was filtered, frozen, and lyophilized (JJ Científica, model LJJ02, São Paulo, Brazil) to obtain the dry extract. An aqueous extraction was intentionally selected because it provides a safe, solvent-free extract suitable for topical oral application while efficiently recovering hydrophilic bioactive compounds, particularly hydrolysable tannins [11].

#### 2.1.3. Exploratory Chromatographic Characterization

The pomegranate extract was characterized using an ultra-high performance liquid chromatography system (UHPLC) coupled to a quadrupole time-of-flight mass spectrometer (Q-TOF) (BRUKER, Q-TOF II^®^, Billerica, MA, USA). Chromatographic separation was performed on a BEH C-18 column (150 mm × 2.1 mm × 1.7 μm) at a flow rate of 560 μL min^−1^ and column temperature of 60 °C. The mobile phases consisted of (A) water containing 0.05% formic acid and (B) acetonitrile. The initial gradient was set at 95:5 (A:B, *v*/*v*) and linearly increased to 30:70 over 30 min. Subsequently, the gradient was rapidly adjusted to 5:95 in 0.1 min (from 36.00 to 36.10 min) and maintained for 4 min. The system was then returned to the initial condition (95:5) for column re-equilibration for 4 min.

For mass spectrometric analysis, the electrospray ionization (ESI) source was operated with a capillary voltage of 4500 eV, nebulizing gas flow of 3 L min^−1^, drying gas flow of 15 L min^−1^, and interface temperature of 250 °C. Nitrogen was used as both the nebulizing and collision gas. Data acquisition was performed in both positive and negative ionization modes. The resulting chromatograms were visualized using Data Analysis 4.3 software (Bruker, Billerica, MA, USA), and the metabolites were putatively annotated using the MassBank of North America (MoNA) spectral library (https://mona.fiehnlab.ucdavis.edu/). Peaks with a minimum intensity threshold of 1000 and an mSigma value of 20 were considered, and possible sodium, ammonium, chloride, and water adducts were included in the analysis.

The chemical profile of cinnamon EO was investigated by gas chromatography coupled to mass spectrometry (GC–MS) using a Thermo Electron Corporation DSQ II system (TLC, Thermo Fisher Scientific Inc., Waltham, MA, USA) equipped with an HP-5 capillary column (30 m × 0.25 mm). The sample (10 μL) was diluted in 1000 μL of dichloromethane, and a 1 μL aliquot was injected using a split ratio of 1:20. The oven temperature was initially set at 40 °C and programmed to increase at 6 °C min^−1^ until reaching 300 °C. The injector and GC–MS interface temperatures were maintained at 250 °C. Helium was used as the carrier gas (0.7 bar; 1 mL min^−1^). Mass spectra were recorded at 70 eV within a mass range of *m*/*z* 50–550 amu.

### 2.2. Antifungal Assay

Antifungal assays were performed according to the method previously described by Dalmagro et al. [12]. *Candida albicans* (ATCC 10231) was initially activated in Brain Heart Infusion (BHI, Acumedia^®^, Lansing, MI, USA) broth and incubated at 25 °C for 48 h. Subsequently, the culture was transferred to BHI agar (Acumedia^®^, Lansing, MI, USA) plates and incubated again at 25 °C for an additional 48 h. After incubation, a fungal suspension was prepared in sterile 0.9% saline solution at a concentration of 1.5 × 10^6^ CFU mL^−1^ and used for subsequent assays.

#### 2.2.1. Minimum Inhibitory Concentration

The minimum inhibitory concentration (MIC) was determined using the serial microdilution method in 96-well microplates, performed in triplicate. The MIC was defined as the lowest concentration of the tested sample capable of visibly inhibiting fungal growth after 48 h of incubation. Turbidity of the culture medium in the wells was used as the criterion for fungal growth.

#### 2.2.2. Plant Synergism

The interaction between the plant-derived substances was assessed using the checkerboard method, performed in triplicate. The initial concentrations used for each sample were 25 mg mL^−1^ for cinnamon EO and 12.5 mg mL^−1^ for pomegranate extract.

Briefly, 100 μL of BHI broth was added to each well of a 96-well microplate. Subsequently, 100 μL of cinnamon EO was added to the wells of column 12. Serial dilutions were performed by transferring 100 μL from column 12 to column 11 and successively until column 2. Next, 100 μL of the pomegranate extract suspension was added to the wells of row A (from 1A to 12A). From this row, 100 μL was sequentially transferred to the subsequent rows until row G. Column 1 served as a control containing only the pomegranate extract, while row H contained only the cinnamon EO, both evaluated individually [12].

Each well then received 5 μL of the *C. albicans* suspension (equivalent to 7.5 × 10^4^ CFU). The plates were incubated at 25 °C for 48 h. Fungal growth was evaluated by visual inspection of turbidity in the culture medium.

The interaction between the tested substances was determined by calculating the Fractional Inhibitory Concentration Index (FICI), according to the following equation:

(1) $$FICI={FIC}_{A}+{FIC}_{B}=\frac{{MIC}_{A}\;in\;combination}{{MIC}_{A}\;separated}+\frac{{MIC}_{B}\;in\;combination}{{MIC}_{B}\;separated},$$

where A and B refer to cinnamon EO and pomegranate extract, respectively.

According to Odds [13], FICI values lower than 0.5 indicate synergistic interaction between the compounds, whereas values greater than 4 indicate antagonism. FICI values ranging from 0.5 to 4 are interpreted as no significant interaction.

### 2.3. Development of the Mucoadhesive System

A mucoadhesive gel was prepared by dispersing 4.0% (*w*/*w*) chitosan (Êxodo Científica, Sumaré, Brazil) in an aqueous solution containing 2% (*w*/*w*) lactic acid under continuous stirring at 25 °C. Subsequently, 5% (*w*/*w*) Tween 80 (Sigma-Aldrich^®^, St. Louis, MO, USA) was incorporated into the gel as a surfactant, following the procedure described by Garcia et al. [14]. After homogenization, 0.195% (*w*/*w*) of each plant-derived material was added to the formulation under constant stirring at 25 °C.

A control formulation, devoid of plant components, was prepared in parallel for comparative purposes. All formulations were prepared in three independent batches and allowed to stand for 24 h to achieve equilibrium prior to physicochemical characterization.

### 2.4. Physical Characterization of the Mucoadhesive System

#### 2.4.1. Organoleptic Evaluation

The organoleptic evaluation was performed to assess the sensory characteristics of the formulation, considering parameters such as color and turbidity. The analysis was conducted at room temperature (~25 °C) under indirect natural lighting using a clean, transparent container. The color of the formulations was evaluated by visual inspection, while turbidity was assessed through direct observation of light transmission through the sample and qualitatively classified according to the degree of clarity (transparent, slightly turbid, turbid, or opaque).

The overall appearance of the gel was also visually examined, considering the homogeneity of the preparation and the possible presence of lumps, bubbles, spots, stains, or phase separation. An ideal formulation was defined as presenting a uniform appearance without sedimentation or physical alterations that could compromise formulation integrity, in accordance with regulatory recommendations [15].

#### 2.4.2. pH Determination

The pH of the formulation was determined using a calibrated digital potentiometer (ION^®^, pHB 500, Araucária, Brazil). The electrode was directly immersed in the sample, and measurements were performed at 25 °C.

#### 2.4.3. Relative Density

Relative density was determined using a previously calibrated 10 mL pycnometer. The sample was introduced into the pycnometer, and the mass was recorded. Density was calculated based on the ratio between the measured mass and the known volume of the pycnometer.

#### 2.4.4. Viscosity

The viscosity of the formulation loaded with plant-derived compounds and the control formulation was determined using a digital Brookfield-type viscometer (MVD-8, Marte^®^, Santa Rita do Sapucaí, Brazil) with a reading accuracy of ± 1.0% and a measurement range of 100 to 2,000,000 mPa·s at 25 °C. Measurements were performed at rotational speeds ranging from 0.3 to 60 rpm using cylindrical spindle number 2, according to the reading limits at maximum rotational speed. After immersion of the spindle in the formulation, the sample was allowed to stand for 5 min before initiating viscosity measurements.

### 2.5. Clinical Trial Design

This pilot study was conducted using a convenience sampling approach involving patients treated at primary healthcare units in the municipality of Umuarama, located in the northwest region of Paraná, Brazil. The study protocol was approved by the Human Research Ethics Committee of Universidade Paranaense (UNIPAR) under approval number 7487137. Initially, 15 patients were screened through clinical examination, and the diagnosis of oral candidiasis was confirmed by a dentist. Eligible participants were users of complete or removable dentures and presented clinical signs and/or symptoms compatible with oral fungal infection, including burning sensation, increased sensitivity, oral pain, and erythematous lesions affecting the gingiva, palate, or tongue.

All individuals received detailed information regarding the study procedures, ensuring full understanding of the study objectives, voluntary participation, and absence of any form of coercion. Of the initially screened participants, 12 agreed to participate and provided written informed consent. Participants were then allocated into two groups through a simple randomization procedure in an open-label, non-controlled design.

Prior to the initiation of treatment, all participants underwent an individualized clinical evaluation, including examination of the oral mucosa, identification and characterization of lesions, assessment of reported symptoms, and evaluation of denture hygiene conditions. Participants were randomly assigned to two groups (*n* = 6 per group) and received the following treatments:

- Gel group: application of the mucoadhesive gel loaded with pomegranate extract and cinnamon EO.
- Nystatin group: administration of oral nystatin suspension (100,000 IU mL^−1^), following the conventional therapeutic regimen commonly used for the treatment of oral candidiasis and denture stomatitis [4].

Exclusion criteria included known allergy to any component of the formulations, concomitant use of topical or systemic antifungal agents, and refusal to sign the informed consent form.

Participants were instructed to remove their dentures prior to each application of the gel or nystatin suspension to allow direct access to the affected areas of the oral mucosa, including the gingiva, palate, or tongue. Participants assigned to the gel group were instructed to apply approximately 0.2 g of the gel (corresponding to a thin layer sufficient to completely cover the lesion) directly onto the affected mucosa using either a clean finger or sterile gauze. The nystatin suspension was administered according to the conventional therapeutic regimen. Both treatments were applied three times daily at 8 h intervals for 14 consecutive days. Participants were instructed not to exceed the prescribed frequency of application and to wait at least 30 min before reinserting their dentures to maximize the contact time between the formulation and the affected tissues.

Treatment adherence was monitored during the scheduled follow-up visits on days 7 and 14 through participant self-report regarding compliance with the prescribed regimen. During these visits, participants received in-person guidance emphasizing the importance of maintaining adequate oral hygiene to support therapeutic success. Hygiene of dentures and remaining teeth was performed using a mechanical cleaning method consisting of brushing with a conventional toothbrush combined with dentifrice or water. This approach was selected because it is simple, accessible, cost-effective, and widely applicable. The use of chemical disinfectants, such as chlorhexidine gluconate, sodium hypochlorite, or alkaline peroxides, was not recommended during the hygiene protocol.

At each follow-up visit, photographic documentation of the lesions was obtained, and the progression of the clinical condition was evaluated, including assessment of lesion size, degree of inflammation, and reduction in pain.

**Figure 1:**
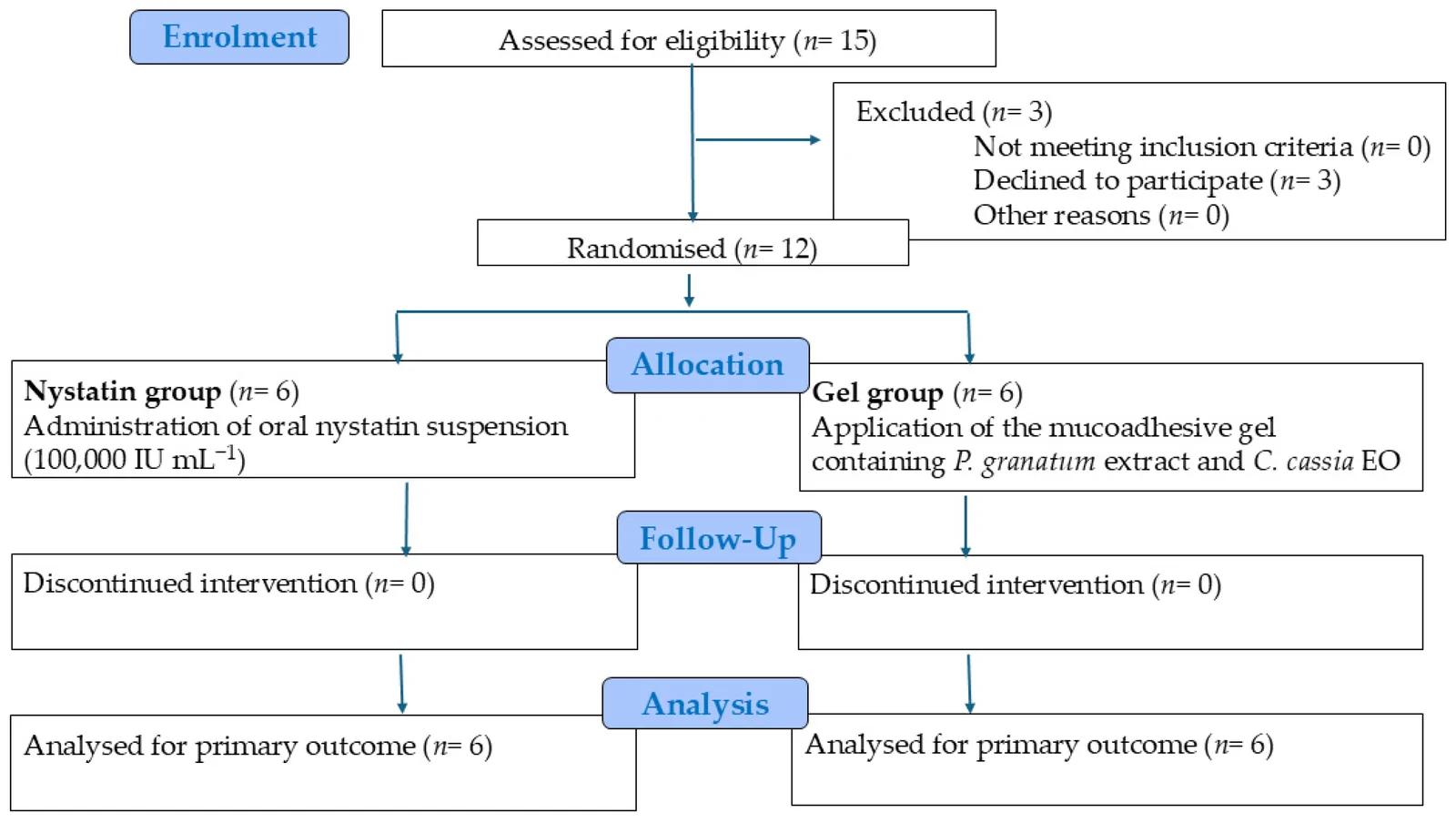
Flow diagram of participant enrollment, allocation, follow-up, and analysis in the two-group randomized trial.

At baseline and on day 14, participants were also interviewed using a Likert-scale questionnaire to assess subjective perceptions related to symptom severity and treatment acceptability. The baseline evaluation allowed identification of the most relevant symptoms perceived by patients, whereas the post-treatment assessment aimed to determine potential changes in the intensity, frequency, or nature of these symptoms. The questionnaire also included items related to the overall acceptability of the treatment or formulation used. The flow diagram illustrating the progression of participants through the phases of the two-group randomized trial is presented in Figure 1.

### 2.6. Statistical Analysis

Experimental data obtained from the two volunteer groups were expressed as mean ± standard error. Comparisons between groups were performed using a paired *t*-test with IBM SPSS Statistics^®^ version 22, adopting a significance level of 5%. As this study was designed as an exploratory pilot trial, no formal a priori sample size calculation was performed. To aid the interpretation of the findings, a post hoc effect size analysis (Hedges’ g), 95% confidence intervals, observed statistical power, and estimated sample sizes for future randomized clinical trials were calculated. Detailed results are provided in Supplementary Table S1.

## 3. Results

This study characterized and evaluated the synergistic effect of cinnamon EO and pomegranate extract against *C. albicans*. Furthermore, it aimed to develop a mucoadhesive system enriched with these natural compounds and to assess its therapeutic potential through a pilot study involving patients with oral candidiasis.

### 3.1. Chemical Characterization and Determination of the Antifungal Activity of Cinnamon Essential Oil and Pomegranate Extract

The antifungal activity of the isolated natural compounds demonstrated a relevant inhibitory effect against *C. albicans*. However, the combination of these materials resulted in a substantial reduction in the concentration required to inhibit fungal growth (Table 1). The FICI confirmed the occurrence of a synergistic effect, indicating that the association of the plant-derived extracts enhances antifungal activity.

Based on this finding, the concentration obtained for the combined extracts was used as a reference for the development of the mucoadhesive formulation intended for oral application. This effect may be attributed, at least in part, to the presence of bioactive compounds in the cinnamon EO (Table 2) and in the pomegranate extract (Table 3) which may act in a complementary manner to inhibit fungal growth.

### 3.2. Physical Characterization of the Formulation

**Figure 2:**
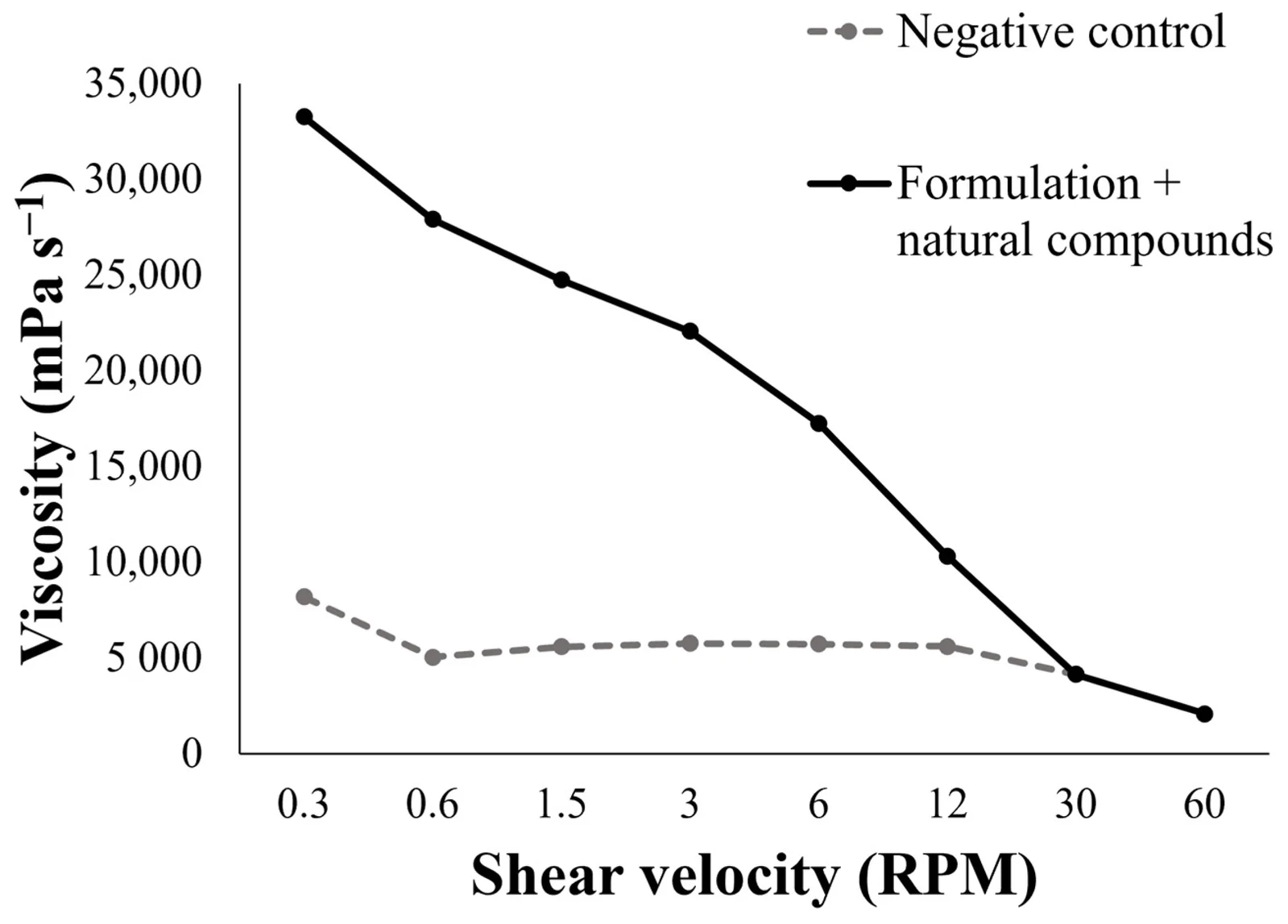
Rheogram of the mucoadhesive formulations.

Table 4 shows a variation in the coloration of the formulations, which can be attributed to the presence of pomegranate extract, characterized by a brown color derived from the fruit peel. In contrast, the formulation without active compounds maintained a slightly yellowish coloration. No significant changes were observed in pH or density; however, the incorporation of the natural compounds resulted in an approximately fourfold increase in the initial viscosity (at 0.3 rpm). Additionally, the formulations exhibited a pseudoplastic flow behavior (Figure 2).

### 3.3. Clinical Evaluation of the Mucoadhesive Formulation and Comparison with Conventional Treatment

The study sample consisted of 12 volunteers equally distributed into two groups (*n* = 6). The sociodemographic and clinical characteristics of the participants (Table 5) indicated a predominance of female participants in both groups (83.3%). In the gel group, the mean age was 65.5 years, whereas in the nystatin group the mean age was 62.3 years, indicating a comparable age profile between groups.

Regarding comorbidities, a higher frequency of arterial hypertension and diabetes mellitus was observed among volunteers in the gel group. Concerning smoking habits, no smokers were identified in the gel group, while one participant in the nystatin group reported being a smoker.

**Figure 3:**
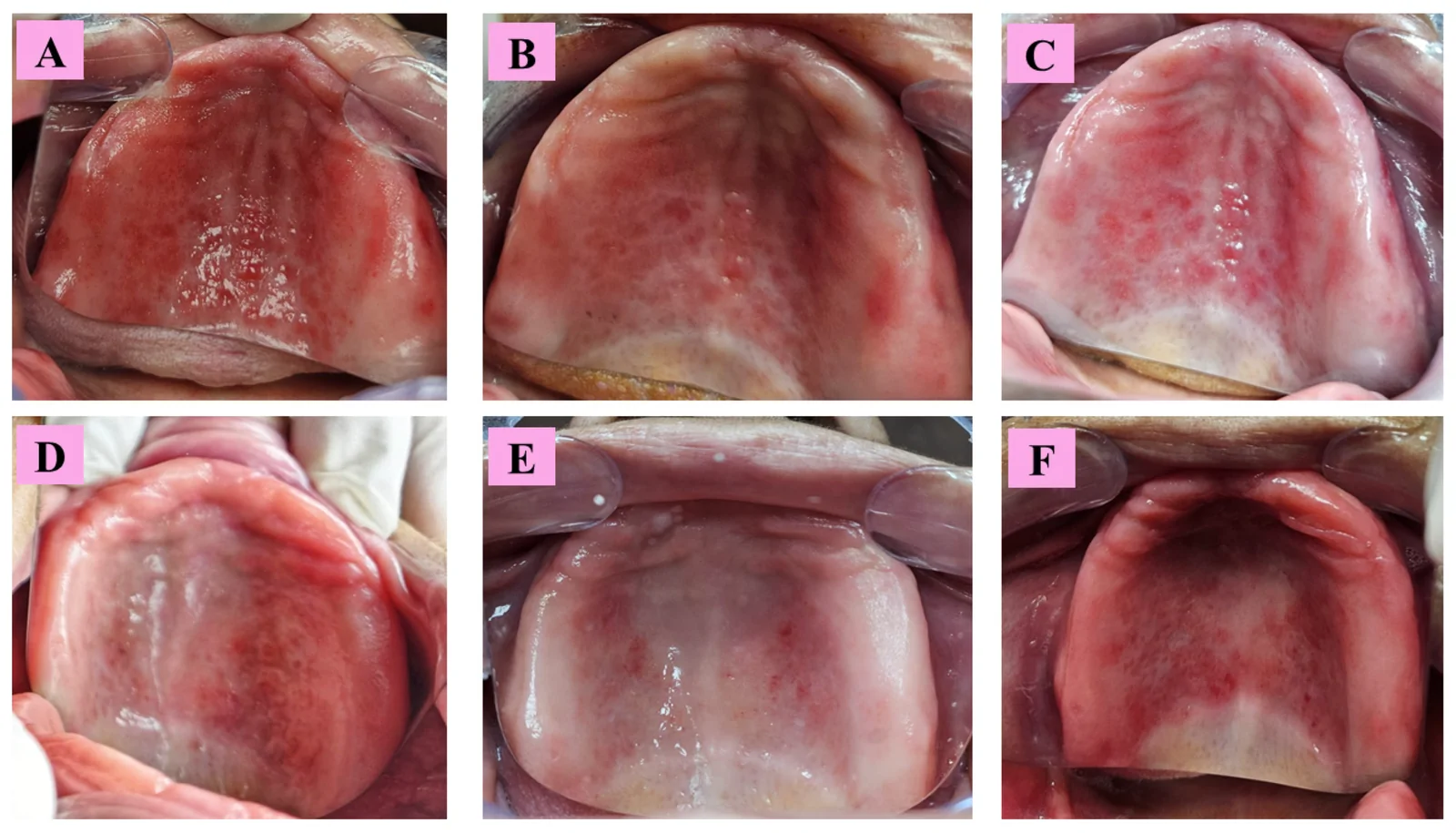
Clinical evolution of the palatal mucosa affected by denture-associated candidiasis at baseline (**A**,**D**), and after 7 (**B**,**E**) and 14 (**C**,**F**) days of treatment. Images (**A**–**C**) correspond to the mucoadhesive gel loaded with pomegranate extract and cinnamon essential oil, whereas images (**D**–**F**) correspond to treatment with nystatin suspension.

Regarding the clinical outcomes observed with the mucoadhesive gel loaded with natural compounds, Figure 3A–C illustrate the sequential evolution of the palatal mucosa affected by erythematous candidiasis associated with complete denture use at three time points (days 1, 7, and 14) following the initiation of treatment.

At baseline examination, the edentulous palatal mucosa exhibited intense and diffuse erythema, predominantly in the region of direct contact with the denture (Figure 3A). In addition, multiple congested papules and nodules were observed, mainly clustered in the central and posterior portions of the hard palate, giving the lesion a granular and hyperplastic appearance characteristic of this clinical presentation of denture-associated candidiasis.

On the seventh day of follow-up, a more favorable clinical condition was observed (Figure 3B). The generalized erythema showed a noticeable reduction, approaching the pale pink coloration typical of healthy mucosa. Although the central papules were still identifiable, they appeared less edematous and less intensely erythematous. Moreover, the clustering pattern became less evident, with a more regular surface, suggesting a positive response to the instituted therapy, both in terms of reduction in fungal burden and modulation of the inflammatory response.

By the fourteenth day, the final clinical record revealed a milder presentation of the alterations. Erythema was restricted to small residual areas, and the previously observed nodular and papillary formations showed marked flattening, resulting in a smoother, more homogeneous mucosal surface that more closely resembled normal tissue.

Overall, the clinical progression over the two-week period demonstrated an incomplete resolution of the inflammatory process; however, the candidiasis presented a more superficial manifestation by the end of the proposed therapeutic approach, with no need for additional therapeutic intervention. This decision was supported by the reports of the volunteers, who remained asymptomatic, without complaints of itching, pain, or irritation at the affected site.

Participants were advised regarding the importance of continuous monitoring of the affected area and the proper maintenance of local hygiene. They were also instructed to return for clinical reassessment if any local changes or new symptoms occurred. These findings reinforce the importance of early diagnosis, appropriate therapy, and the elimination of predisposing factors for the effective management of denture-associated candidiasis.

Figure 3D–F sequentially illustrate the evolution of the lesion and its response to conventional treatment with nystatin suspension. In the initial image (Figure 3D), the clinical presentation is comparable to that observed at baseline in the gel-treated patient. The upper palatal mucosa, in a completely edentulous area, exhibited intense and diffuse erythema restricted to the region of contact with the denture base. A notable feature was the presence of multiple congested and sessile papules and nodules located in the central region of the hard palate, producing a granular and hyperplastic surface texture. This clinical manifestation reflects the initial stage of an active chronic fungal infection and served as the reference point for evaluating the therapeutic effectiveness of nystatin.

By day 7, a noticeable improvement in the inflammatory condition was observed (Figure 3E). The diffuse erythema showed substantial regression, with the mucosa recovering a paler pink coloration, indicating a reduction in hyperemia and fungal burden. The most relevant improvement was associated with the regression of the central hyperplastic papules, which appeared significantly less edematous and flatter, losing the prominence and congestion previously observed.

Despite this intermediate therapeutic response, the evaluation at day 14 revealed a recurrence of intense and diffuse erythema in the denture-contact area (Figure 3F). Additionally, the central region of the palate again displayed a reddish dotted and clustered pattern, suggesting areas of vascular congestion and possible reactivation of the hyperplastic papules. Overall, the clinical evolution over the two-week period indicated incomplete resolution and a recurrent pattern of the inflammatory process. Notably, in three of the six patients (50.0%), there was significant persistence of oral lesions and associated symptoms, such as burning sensation and difficulty swallowing, which required the introduction of complementary systemic therapy.

At baseline and at the end of the treatment period, symptom assessment was performed using a questionnaire composed of eight items, each with five response options based on an ordinal Likert-type scale ranging from 1 to 5. In this scale, the values represent the frequency and intensity of symptoms, varying from “none” (1) to “very intense” (5), including clinical manifestations such as hyperplastic papules. This approach allowed a standardized evaluation of patients’ perceived symptoms, enabling comparison between the assessed groups (Table 6) at baseline and at the end of the follow-up period.

Overall, although variations in the mean scores of oral candidiasis symptoms were observed over the 14-day treatment period, no statistically significant differences were detected for any of the evaluated items between baseline and final assessments in either group (*p* > 0.05). These findings indicate that, from a statistical perspective, neither intervention resulted in a significant reduction in self-reported symptoms during the analyzed period.

Nevertheless, a descriptive trend toward a more consistent clinical improvement was observed in the gel group, particularly for inflammatory symptoms such as oral redness/inflammation (Q3), as well as a slight reduction in dry mouth (Q5) and overall oral sensitivity (Q8). In contrast, the nystatin group showed greater stability in symptom scores, with minor fluctuations and a slight increase in some inflammatory symptoms.

Considering the small sample size (*n* = 6 per group), the study may not have had sufficient statistical power to detect intra- or intergroup differences, thus characterizing it as an exploratory pilot study. Therefore, these results should be interpreted with caution, as they suggest clinical tendencies rather than providing definitive conclusions.

Another relevant aspect concerns patient acceptability of the treatments. To assess this parameter, a questionnaire was administered on day 14 of treatment, consisting of ten items, each with five response options based on an ordinal Likert-type scale ranging from 1 to 5, in which the values represent satisfaction and acceptability, varying from “dissatisfied” (1) to “fully satisfied” (5) (Table 7). The results indicated a positive evaluation of both treatments regarding acceptability and satisfaction after 14 days of use.

A statistically significant difference favoring the gel formulation was observed for the following items: ease of application (Q9; *p* = 0.049), absence of irritation (Q11; *p* = 0.008), and lack of interference with oral functions (Q15; *p* = 0.003), indicating superior tolerability and greater functional comfort compared with nystatin. In addition, the gel showed higher mean scores, although not reaching statistical significance, for adhesion to the oral mucosa, ease of removal, perceived retention, and overall satisfaction. Regarding the intention for future use (Q16) and willingness to recommend the treatment to others (Q17), both treatments received similarly high scores.

Thus, although both treatments demonstrated comparable clinical effectiveness in reducing symptoms, the gel formulation showed advantages in terms of acceptability, comfort, and patient experience, which may represent a relevant therapeutic benefit from a functional perspective and could potentially favor better treatment adherence in studies involving larger sample sizes.

## 4. Discussion

Epidemiological studies indicate a significant increase in the use of medicinal plants and herbal formulations, reflecting their gradual incorporation into healthcare systems as well as advances in scientific research aimed at developing standardized and safe natural products [16]. In this context, the exploration of plant-derived compounds as adjuvant therapeutic strategies has gained relevance, particularly for recurrent conditions such as oral candidiasis. This infection remains a frequent clinical problem, characterized by high recurrence rates, emerging antifungal resistance, and negative effects on oral comfort and quality of life, especially among denture wearers.

In the present study, the combination of cinnamon EO and aqueous pomegranate extract exhibited a synergistic antifungal effect against *C. albicans*, resulting in greater inhibition of fungal growth than either natural product used individually. Importantly, this interaction was experimentally confirmed using the checkerboard broth microdilution assay, which yielded a Fractional Inhibitory Concentration Index (FICI) of 0.250. According to internationally accepted interpretative criteria, FICI values ≤ 0.50 indicate a synergistic interaction, thereby demonstrating that the enhanced antifungal activity observed resulted from true pharmacological synergism rather than a merely additive effect. The observed interaction suggests that combining bioactive compounds with distinct mechanisms of action may enhance antifungal efficacy while potentially reducing the effective concentrations required for each component.

The antifungal effect of the pomegranate extract can be largely attributed to the high concentration of hydrolysable tannins in the fruit peel, particularly punicalagin and related phenolic derivatives. These compounds interact with proteins and polysaccharides of the fungal cell wall, promoting macromolecule precipitation, altering membrane permeability, and inhibiting enzymes involved in microbial metabolism [17,18]. Likewise, the antifungal activity of cinnamon is primarily associated with cinnamaldehyde, which disrupts fungal membrane integrity, leading to leakage of intracellular constituents and mitochondrial dysfunction. The complementary action of these bioactive compounds on multiple cellular targets likely explains the synergistic interaction observed in the present study, supporting previous reports that combinations of natural products may enhance antifungal efficacy while reducing the likelihood of resistance development [19,20,21].

Interestingly, despite the marked differences in the phytochemical composition of the aqueous pomegranate extract and cinnamon EO, both natural products exhibited identical MIC values against *C. albicans*. This finding suggests that, under the experimental conditions employed, both phytochemical matrices displayed comparable overall antifungal potency. It should be emphasized that MIC reflects the overall biological activity of the complete phytochemical matrix rather than the concentration or potency of individual constituents. Consequently, distinct phytochemical profiles and mechanisms of action may converge to produce similar inhibitory concentrations while acting through different cellular targets.

Oral candidiasis is strongly associated with several predisposing factors, including immunosuppression, prolonged antibiotic therapy, oncological treatments, and systemic debilitating conditions [22]. In denture users, additional factors such as biofilm formation, inadequate hygiene, prosthesis structure, and autoinoculation contribute to persistent colonization by *Candida* species [2]. Lifestyle habits and systemic conditions also play a relevant role. Smoking may impair tissue healing and contribute to alterations in salivary flow, whereas several medications can induce xerostomia. The reduction in salivary secretion compromises the natural cleansing and antimicrobial functions of saliva, thereby favoring microbial colonization and increasing the risk of oral candidiasis [23,24,25]. These factors are further compounded by the increasing antifungal resistance reported among *Candida* species, particularly in aging populations and individuals exposed to prolonged antifungal therapy [26,27].

Conventional management of oral candidiasis generally involves topical or systemic antifungal agents, with nystatin being widely used due to its broad spectrum of activity, favorable safety profile, and low cost [28]. Nevertheless, complete clinical remission is not always achieved, particularly in persistent infections or in cases of reduced fungal susceptibility. Consequently, complementary therapeutic strategies have attracted increasing attention, especially those based on natural products with multifactorial mechanisms of action and potential anti-inflammatory properties [29,30].

From a pharmaceutical perspective, the oral mucosa represents a favorable route for local drug delivery due to its accessibility, vascularization, and regenerative capacity. Mucoadhesive systems have been widely investigated as platforms capable of prolonging the residence time of active compounds at the site of infection, thereby enhancing local bioavailability and therapeutic effectiveness [31,32]. In the present study, the developed gel exhibited high viscosity at rest and pseudoplastic flow behavior, rheological characteristics considered desirable for mucoadhesive formulations. High viscosity contributes to structural stability and retention at the application site, whereas pseudoplastic behavior facilitates spreading during application, while allowing the formulation to rapidly recover its viscosity after application, thereby favoring prolonged mucosal retention [12]. Such properties are particularly advantageous in the oral cavity, where constant muscular movement and salivary flow can compromise the retention of topical formulations [33,34].

Although the pH of the formulation was slightly lower than the physiological pH of the oral mucosa, no irritation or discomfort was reported by patients, suggesting satisfactory biocompatibility. This observation may be explained by the buffering capacity of saliva and the physiological tolerance of the oral mucosa to moderate pH variations in topical formulations [35].

In the pilot clinical evaluation, both treatments produced comparable reductions in oral candidiasis symptoms over the 14-day treatment period, with no statistically significant differences between groups. This result is consistent with previous reports indicating that topical antifungal therapies can promote gradual symptom improvement in mild to moderate infections. However, a trend toward greater improvement was observed in the gel group, particularly in inflammatory symptoms such as redness and local discomfort, as well as slight reductions in xerostomia and oral sensitivity.

These findings may be partly explained by the protective properties of the mucoadhesive system. In addition to delivering antifungal agents, the gel forms a bioadhesive layer over the mucosa, which may reduce mechanical friction between the denture and oral tissues, minimize microtrauma, and help maintain local hydration. Previous studies have demonstrated that mucoadhesive systems can act not only as drug delivery platforms but also as protective barriers for inflamed mucosa, contributing to improved comfort and tissue repair [36,37,38].

Patient acceptability also emerged as an important aspect of the developed formulation. The gel demonstrated advantages in parameters such as ease of application, absence of irritation, and minimal interference with oral functions. These findings are consistent with evidence indicating that rheological behavior and mucoadhesive properties play a key role in patient experience and treatment adherence [12,33]. Higher scores for parameters such as mucosal adhesion, perceived retention, and overall satisfaction reinforce the importance of acceptability as a determinant of therapeutic success, particularly in antifungal therapies where inadequate adherence represents a major cause of recurrence [39].

An additional relevant observation was that none of the patients treated with the gel required complementary therapy during the evaluation period. Although the limited number of participants prevents definitive conclusions, this finding suggests that prolonged residence time and mucosal protection may contribute to improved local therapeutic outcomes [31,32]. It should be emphasized that the purpose of this study was not to replace conventional antifungal therapies, but to explore a complementary strategy for the management of oral candidiasis.

Although the present study included a limited number of participants, which may have reduced the statistical power to detect differences between treatments, post hoc effect size analysis demonstrated large to very large effects for the principal acceptability outcomes (Supplementary Table S1). In particular, the absence of irritation (Q11; Hedges’ *g* = 1.76; 95% CI: 0.39–3.13) and no interference with oral functions (Q15; Hedges’ *g* = 2.13; 95% CI: 0.66–3.60) exhibited very large effect sizes, indicating clinically meaningful advantages of the mucoadhesive gel over nystatin regarding local tolerability and preservation of oral functions. From a clinical perspective, these characteristics are particularly relevant for topical oral therapies, as local irritation, discomfort, and interference with speech or swallowing may negatively affect patient adherence and compromise treatment effectiveness. Therefore, despite the exploratory nature of this pilot study, the large effect sizes observed for these outcomes support the clinical relevance of the developed formulation and reinforce its potential as a promising adjunctive therapy for denture-associated oral candidiasis. Furthermore, the effect size estimates and corresponding sample size calculations generated from these preliminary data provide a valuable basis for designing future adequately powered randomized clinical trials.

Taken together, these findings suggest that the developed mucoadhesive phytotherapeutic gel represents a promising adjunctive strategy for the management of denture-associated oral candidiasis. By combining synergistic antifungal activity with prolonged mucosal residence, high patient acceptability, and protective effects on inflamed oral tissues, the formulation may contribute to improving local treatment outcomes while complementing conventional antifungal therapy.

Beyond its application as a therapeutic mucoadhesive gel, the demonstrated synergistic activity of the combination of pomegranate extract and cinnamon EO opens new perspectives for the development of preventive oral healthcare products, including herbal toothpastes and mouthwashes aimed at reducing microbial colonization and denture-associated biofilm formation. Future studies should also investigate the in vitro release kinetics of cinnamaldehyde and punicalagin, their permeation and retention within the oral mucosa, and establish in vitro–in vivo correlations to optimize formulation parameters, including viscosity. Furthermore, the development of these alternative pharmaceutical systems should include investigations of formulation stability, antimicrobial efficacy against multispecies biofilms, and long-term clinical performance.

Overall, this exploratory pilot study provides the first evidence that the synergistic combination of pomegranate extract and cinnamon EO can be successfully incorporated into a mucoadhesive drug-delivery system with favorable physicochemical properties, clinically relevant antifungal activity, and promising patient acceptability. To the best of our knowledge, this is the first study to describe both the pharmaceutical development and preliminary clinical evaluation of this phytotherapeutic combination for the management of denture-associated oral candidiasis. Although these findings should be confirmed in larger randomized controlled clinical trials, the present results establish a solid foundation for the development of innovative, low-cost phytotherapeutic strategies as adjunctive therapies for oral fungal infections. Collectively, these future investigations will provide the scientific basis for the rational optimization of this drug-delivery platform, enabling refinement of formulation design, validation under appropriately controlled conditions, and expansion of its clinical applications in oral healthcare.

## Figures and Tables

**Table 1:** Minimum inhibitory concentrations (MICs) of crude pomegranate extract, cinnamon essential oil, and their combination against *C. albicans*, as determined by broth microdilution and checkerboard assays.

Natural Product	MIC (mg mL^−1^) *
Pomegranate extract	1.562 ± 0.001
Cinnamon essential oil	1.562 ± 0.002
Pomegranate extract + Cinnamon essential oil	0.195 + 0.195
Fractional inhibitory concentration index (FICI)	0.250 ± 0.000

* Results are expressed as mean ± standard error (*n* = 3). MIC values correspond to the concentrations of the crude aqueous pomegranate extract and the whole cinnamon essential oil tested. For the combination, the reported values represent the final concentration of each individual component in the checkerboard assay at the point of complete growth inhibition.

**Table 2:** Chemical composition and relative percentage (%) of cinnamon essential oil by GC-MS.

Retention Time (min)	Compound	Percentage (%) ^1^
8.22	Styrene	0.139
10.37	Camphene	0.027
12.86	ϒ-Terpinene	0.004
13.44	p-Cymene	0.045
13.62	D-Limonene	0.032
13.74	Eucalyptol	0.020
15.14	Acetophenone	0.130
17.68	α-Camphonelal	0.010
19.82	Terpinen-4-ol	0.040
24.26	Trans-Cinnamaldehyde	74.579
26.39	Hydrocinnamic acid	0.173
27.05	Eugenol	0.040
27.73	Copaene	0.877
28.87	Caryophyllene	0.029
30.05	Coumarin	3.846
30.29	Cinnamyl acetate	4.725
31.37	ϒ-Muurolene	0.276
32.19	α-Muurolene	0.169
32.46	β-Bisabolone	0.226
33.53	2-Methoxycinnamaldehyde	14.535
34.05	14-Hydroxycaryophyllene	0.039
35.60	Humulene epoxide	0.026
38.54	Caryophyllene oxide	0.013

^1^ Relative to the total GC-MS peak area.

**Table 3:** Phytochemical identification of pomegranate extract by UHPLC–MS.

RT ^1^	Compound	MF ^2^	Positive (*m*/*z*)			Negative (*m*/*z*)			Class
			Theor ^3^ *m*/*z*	Exper ^4^ *m*/*z*	Area (%)	Theor ^3^ *m*/*z*	Exper ^4^ *m*/*z*	Area (%)	
1.23	Mannitol	C_6_H_14_O_6_	-	-	-	181.071	181.0714	25.73	Sugar
1.66	Malic acid	C_4_H_6_O_5_	-	-	-	133.0137	133.0139	7.36	Organic acid
2.25	Citric acid	C_6_H_8_O_7_	193.0342	193.0336	4.91	191.0194	191.0192	8.21	Organic acid
2.54	Punicalin	C_34_H_22_O_22_	-	-	-	781.0518	781.0514	6.54	Tannin
2.68	Gallic acid	C_7_H_6_O_5_	171.0287	171.0284	3.50	169.0142	169.0137	3.93	Phenolic acid
3.49	Epigallocatechin	C_15_H_14_O_7_	307.0812	307.0808	39.12	305.0674	305.0661	7.89	Flavonoid
4.15	Punicalagin	C_48_H_28_O_30_	-	-	-	1083.0581	1083.0633	19.69	Tannin
4.41	Procyanidin B2	C_30_H_26_O_12_	-	-	-	577.1359	577.1342	1.32	Proanthocyanidin
5.13	Epicatechin	C_15_H_14_O_6_	291.0870	291.0857	29.12	289.0717	289.0715	5.69	Flavonoid
8.94	Rutin	C_27_H_30_O_16_	-	-	-	609.1461	609.1444	0.23	Flavonoid
9.07	Isoquercitrin	C_21_H_20_O_12_	465.1034	465.1040	2.46	463.0882	463.0879	0.48	Flavonoid
9.35	Ellagic acid	C_14_H_6_O_8_	303.0135	303.0134	20.89	300.9990	300.9983	11.86	Polyphenol
9.66	Nicotiflorine	C_27_H_30_O_15_	-	-	-	593.1511	593.1501	0.46	Flavonoid
9.72	Kaempferol-3-O-glucoside	C_21_H_20_O_11_	-	-	-	447.0934	447.0931	0.64	Flavonoid

^1^ Retention time (minutes); ^2^ Molecular formula; ^3^ Theorical (*m*/*z*); ^4^ Experimental (*m*/*z*).

**Table 4:** Appearance, pH, density, and viscosity of mucoadhesive formulations without active compounds (negative control) and those containing pomegranate extract and cinnamon essential oil.

Parameter	Negative Control	Formulation + Natural Products
Color	Yellowish	Light brown
Appearance	Opaque	Opaque
pH	5.13 ± 0.01	5.30 ± 0.01
Density (g mL^−1^)	1.05 ± 0.00	1.02 ± 0.00
Viscosity at 0.3 RPM (mPa s^−1^)	8178.2	33,262.7

Results are presented as mean ± standard error (*n* = 3).

**Table 5:** Sociodemographic and clinical characteristics of the volunteers.

Variables	Gel Group (*n* = 6)	Nystatin Group (*n* = 6)
Gender:		
Female	5	5
Male	1	1
Mean age (years) ± Standard deviation	65.6 ± 9.53	62.3 ± 7.97
Comorbidities:		
Arterial hypertension	5	3
Diabetes Mellitus	3	2
No comorbidities	1	2
Smoking habits	0	1

**Table 6:** Evolution of oral candidiasis symptoms during treatment (Questions 1–8).

Question	Symptomatology	Gel Group (*n* = 6)		Nystatin Group (*n* = 6)		Paired *t*-Test ^1^
		Baseline	14th Day	Baseline	14th Day	
Q1	Sensitivity to acidic or hot foods	2.33 ± 0.49	1.67 ± 0.33	1.67 ± 0.67	1.67 ± 0.67	NS
Q2	Pain or burning during speaking or eating	1.17 ± 0.17	1.17 ± 0.17	1.67 ± 0.49	1.33 ± 0.33	NS
Q3	Oral erythema/inflammation	2.83 ± 0.65	1.33 ± 0.33	1.83 ± 0.40	2.33 ± 0.42	NS
Q4	Oral burning sensation	2.17 ± 0.75	1.83 ± 0.54	1.33 ± 0.33	1.67 ± 0.42	NS
Q5	Xerostomia (dry mouth)	2.50 ± 0.81	2.33 ± 0.71	3.00 ± 0.52	3.33 ± 0.58	NS
Q6	Dysphagia (difficulty swallowing)	1.17 ± 0.49	1.33 ± 0.33	2.17 ± 0.65	2.17 ± 0.65	NS
Q7	Dysgeusia (metallic or unusual taste)	1.50 ± 0.50	1.50 ± 0.50	1.33 ± 0.21	1.67± 0.49	NS
Q8	General oral sensitivity	2.67 ± 0.58	2.17 ± 0.48	1.50 ± 0.50	1.67 ± 0.67	NS

Data are presented as mean ± standard error of the mean (SEM) for patients in each group. ^1^ NS = not significant.

**Table 7:** Patient acceptability of the treatment assessed by questionnaire (Questions 9–18).

Question	Evaluation	Gel Group (*n* = 6)	Nystatin Group (*n* = 6)	*p*-Value ^1^
Q9	Ease of application and packaging	4.50 ± 0.22	4.00 ± 0.00	0.049
Q10	Palatability	2.83 ± 0.48	3.67 ± 0.42	NS
Q11	Absence of irritation	4.83 ± 0.17	3.33 ± 0.42	0.008
Q12	Adhesion to the oral mucosa	4.33 ± 0.33	3.50 ± 0.34	NS
Q13	Ease of removal	4.50 ± 0.50	3.67 ± 0.21	NS
Q14	Perceived retention at the application site	4.00 ± 0.52	3.67 ± 0.21	NS
Q15	No interference with oral functions	5.00 ± 0.00	3.67 ± 0.33	0.003
Q16	Willingness for future use	4.83 ± 0.17	4.17 ± 0.41	0.018
Q17	Willingness to recommend to others	4.83 ± 0.17	4.17 ± 0.41	0.018
Q18	Overall satisfaction	4.67 ± 0.21	4.33 ± 0.21	NS

Data are presented as mean ± standard error of the mean (SEM) for patients in each group. ^1^ NS = not significant.

## Data Availability

The datasets generated and/or analyzed during the current study are available within the manuscript. Additional information and supporting data are available from the corresponding author upon request.

## Supplementary Materials

The following supporting information can be downloaded at: https://www.mdpi.com/article/10.3390/pharmaceutics18080914/s1, Table S1: Post hoc effect size analysis, observed statistical power, and estimated sample size for future randomized clinical trials based on the primary acceptability outcomes. File S1: Representative clinical photographs showing the progression of denture-associated oral candidiasis lesions at baseline (A), day 7 (B), and day 14 (C). Images are presented sequentially for participants treated with the developed mucoadhesive gel, followed by participants treated with nystatin suspension.
