# Supplementary material for: Mucoadhesive Gel Based on Cinnamomum cassia Essential Oil and Punica granatum Extract for Oral Candidiasis: A Pilot Study of Plant Synergism
Source: Pharmaceutics. 2026 Jul 24;18(8):914. doi: 10.3390/pharmaceutics18080914 (PMC13516971; doi:10.3390/pharmaceutics18080914)

Supplementary File S1. Representative clinical photographs showing the progression of denture-associated oral candidiasis lesions at baseline (A), day 7 (B), and day 14 (C). Images are presented sequentially for participants treated with the developed mucoadhesive gel, followed by participants treated with nystatin suspension.

Mucoadhesive gel: A B C

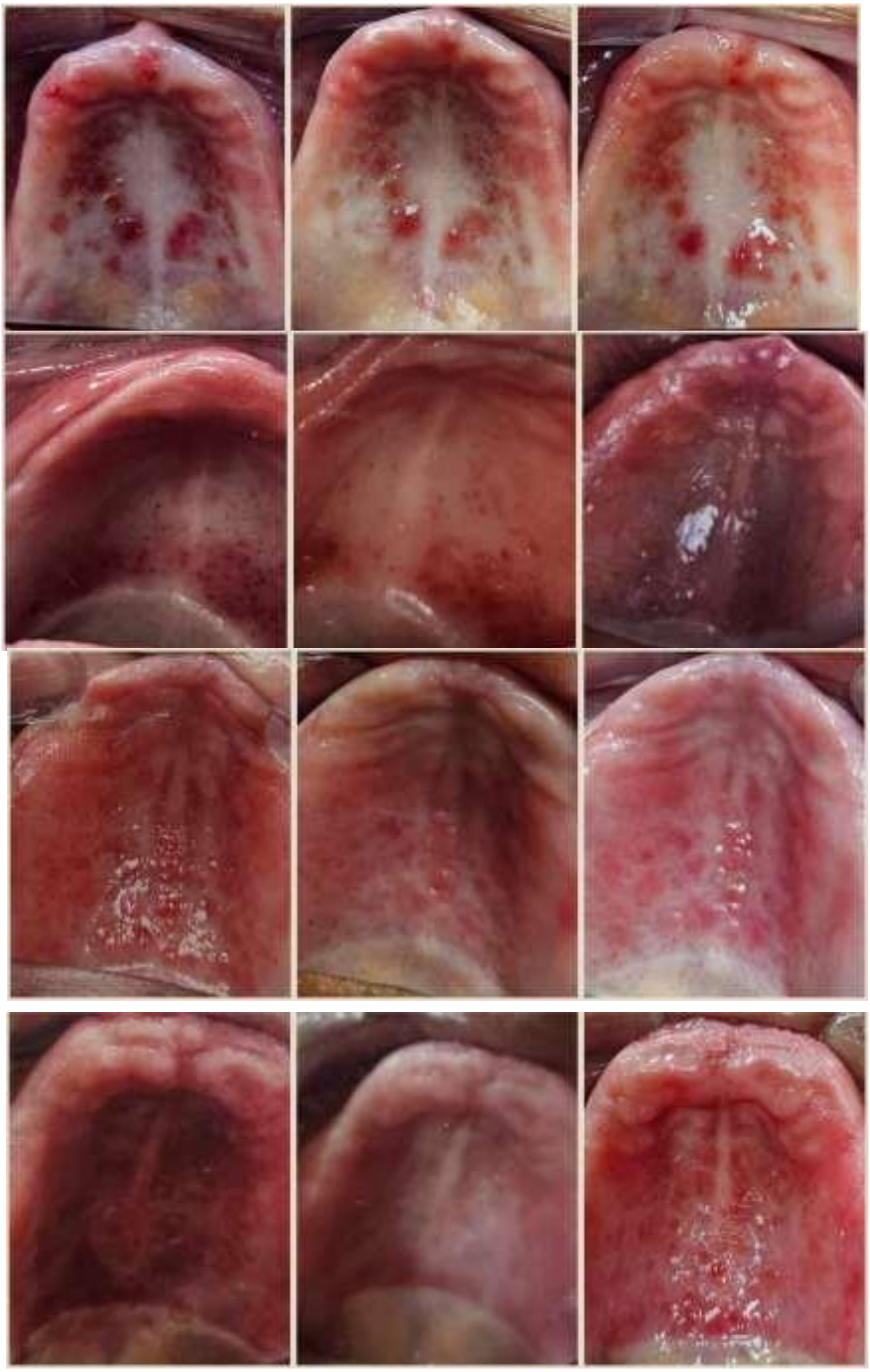

Mucoadhesive gel: A B C

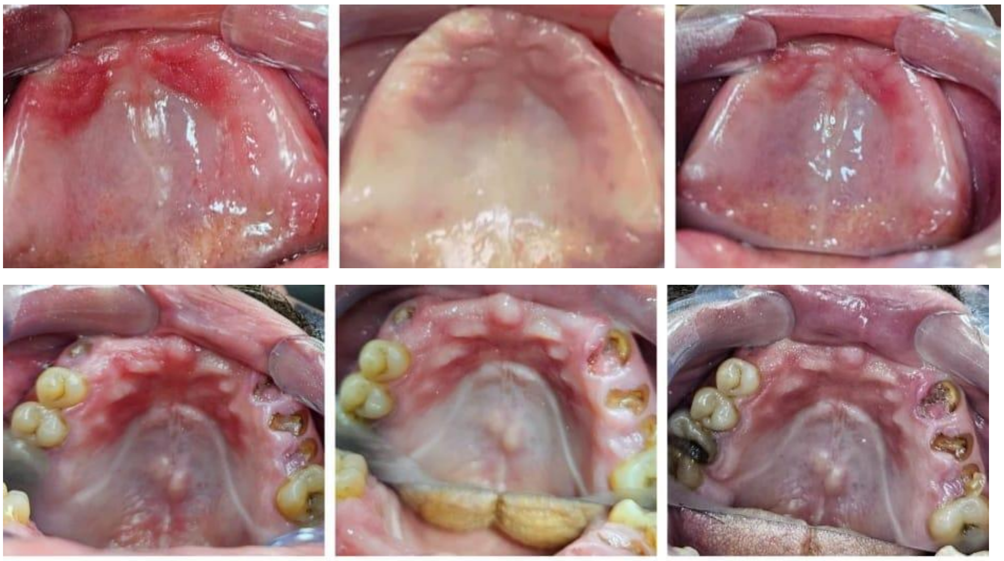

Nystatin suspension: A B C

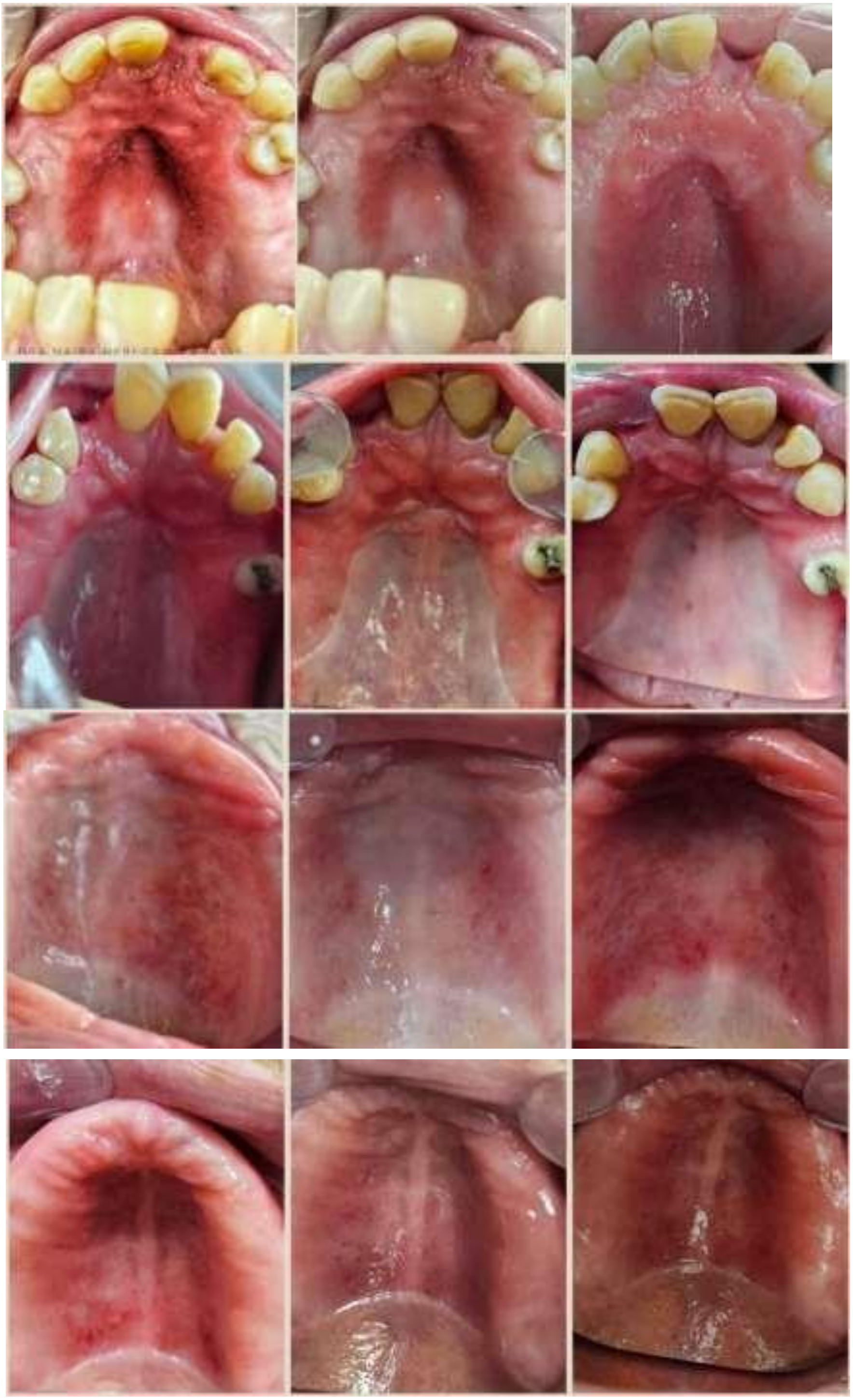

Nystatin suspension: A B C

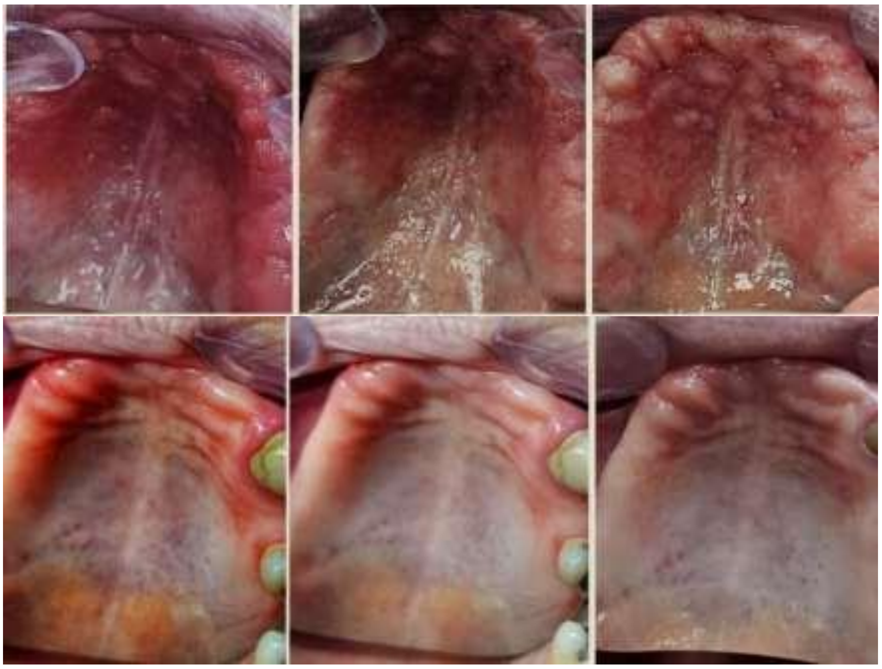

Supplement: Supplementary file 1 [file pharmaceutics-18-00914-s001.zip › Supplementary File S1.pdf]
