# Supplementary material for: Mucoadhesive Gel Based on Cinnamomum cassia Essential Oil and Punica granatum Extract for Oral Candidiasis: A Pilot Study of Plant Synergism
Source: Pharmaceutics. 2026 Jul 24;18(8):914. doi: 10.3390/pharmaceutics18080914 (PMC13516971; doi:10.3390/pharmaceutics18080914)
Supplement: Supplementary file 1 [file pharmaceutics-18-00914-s001.zip › Supplementary Table S1.pdf]

Table S1. Post hoc effect size analysis, observed statistical power, and estimated sample size for future randomized clinical trials based on the primary acceptability outcomes.

Outcome	Hedges' *g*	95% Confidence Interval	Effect Size Interpretation	Observed Power (%)	Estimated Sample Size per Group		
					80% Power	90% Power	95% Power
Q9 – Ease of application	1.19	−0.05 to 2.44	Large	52.4	11	14	17
Q10 – Ease of removal	−0.70	−1.87 to 0.47	Moderate	22.0	29	38	47
Q11 – Absence of irritation	1.76	0.39 to 3.13	Very large	84.6	6	7	9
Q12 – Adhesion to the oral mucosa	0.93	−0.27 to 2.13	Large	35.2	17	22	27
Q13 – Perceived retention	0.82	−0.37 to 2.01	Large	28.5	21	28	34
Q14 – Product taste	0.32	−0.82 to 1.46	Small	8.4	133	178	219
Q15 – No interference with oral functions	2.13	0.66 to 3.60	Very large	94.9	5	6	7
Q16 – Willingness to use the product again	1.51	0.20 to 2.82	Very large	72.2	7	9	11
Q17 – Willingness to recommend the product	1.51	0.20 to 2.82	Very large	72.2	7	9	11
Q18 – Overall satisfaction	0.60	−0.57 to 1.76	Moderate	17.3	39	52	64

Hedges' *g* values were calculated using the pooled standard deviation with correction for small sample sizes. Effect sizes were interpreted according to Cohen's criteria as small (0.20–0.49), moderate (0.50–0.79), large (0.80–1.19), and very large (≥1.20). Estimated sample sizes were calculated assuming a two-sided significance level of $\alpha = 0.05$, equal allocation between groups (1:1), and statistical powers of 80%, 90%, and 95%, respectively. Observed statistical power is presented for exploratory purposes only and should be interpreted with caution given the pilot nature of the study.
